# Integrated metabolome and transcriptome analysis provides insights into anthocyanin biosynthesis in *Portulaca oleracea*

**DOI:** 10.1186/s12870-025-07790-2

**Published:** 2025-11-27

**Authors:** Mingzhao Zhu, Hanying Wu, Yiqian Fu, Linlin You, Caiyun Yang, Ran Zhao, Xiangyang Han

**Affiliations:** 1 State Key Laboratory of Vegetable Biobreeding, Beijing Key Laboratory of Crop Molecular Design and Intelligent Breeding, Key Laboratory of Biology and Genetics Improvement of Horticultural Crops (North China), National Engineering Research Center for Vegetables, Beijing Vegetable Research Center, Beijing Academy of Agricultural and Forestry Sciences, Beijing, 100097 China; 2 Key Laboratory of Systematic and Evolutionary Botany, Institute of Botany, The Chinese Academy of Sciences, Beijing, 100093 China; 3 State Key Laboratory of Plant Diversity and Specialty Crops, Institute of Botany, The Chinese Academy of Sciences, Beijing, 100093 China; 4 China National Botanical Garden, Beijing, China

**Keywords:** *Portulaca oleracea*, Anthocyanin, Metabolome, GST

## Abstract

**Background:**

Pigment synthesis pathways in Caryophyllales have long been debated, with uncertainties surrounding the biosynthesis and regulation of anthocyanins in representative species. *Portulaca oleracea* provides a suitable model to clarify these mechanisms, which is valuable for understanding pigment metabolism and informing molecular breeding within Caryophyllales.

**Results:**

Using a systematic multi-omics approach, we elucidated the anthocyanin biosynthesis mechanism in *P. olerace*a. Peonidin-3-O-glucoside was identified as the predominant anthocyanin component, and its abundance closely correlated with organ-specific coloration. We identified twenty-one key structural genes in the anthocyanin pathway (including *CHS*, *DFR*, *ANS*, and *UFGT*) and eleven transcription factors regulating anthocyanin accumulation, all showing distinct tissue-specific expression patterns. Notably, although the canonical anthocyanin transporter TT19 is absent in *P. olerace*a, a GST family gene (*evm.TU.LG12.1168*) was strongly associated with anthocyanin accumulation, suggesting a potential role in vacuolar transport.

**Conclusions:**

This work establishes a systematic molecular framework for anthocyanin biosynthesis and regulation in *P. oleracea*, highlights peonidin-3-O-glucoside as the primary pigment determinant of organ coloration, and proposes a GST-mediated transport mechanism in the absence of TT19. These findings offer theoretical insights for pigment metabolism research and provide a foundation for molecular breeding strategies across Caryophyllales.

**Supplementary Information:**

The online version contains supplementary material available at 10.1186/s12870-025-07790-2.

## Background

In nature, the coordinated action of diverse plant pigments produces a striking array of colors in flowers, leaves, fruits, stems, etc. Among these pigments, betalains—which confer red and purple hues—are predominantly found in members of the Amaranthaceae family, including beets, amaranth, and cacti, but rarely occur in other plant lineages [32]. Carotenoids contribute to yellow, orange, and red colors, commonly seen in crops like carrots, pumpkins, and corn [40]. In contrast, anthocyanins are the main pigments imparting red, blue, and purple colors to plant organs. They are broadly distributed in a variety of plants, including grapes, blueberries, and red cabbage, and play important roles in antioxidation and stress responses [1, 12]. The distribution and function of diverse plant pigments not only create the vibrant color landscapes observed in nature but also support crucial physiological and ecological processes, offering valuable insights into plant diversity and adaptive strategies.

Among plant-derived natural pigments, anthocyanins stand out as a key contributor to plant color diversity due to their widespread distribution and rich color expression. The chromatic characteristics of anthocyanins are regulated by the pH of cellular fluids, enabling a spectrum of colors ranging from red and purple to blue [24]. Based on differences in glycoside structures, anthocyanins are primarily classified into six major types: peonidin, cyanidin, pelargonidin, delphinidin, petunidin, and malvidin [34]. From a plant physiological perspective, anthocyanins exhibit multiple biological functions: attracting pollinators through vibrant coloration to enhance reproductive success [3]; acting as photoprotective agents that absorb excess light energy, thereby mitigating photooxidative damage [26]; and participating in stress resistance and defense mechanisms. For human health, anthocyanins are particularly notable for their antioxidant and anti-inflammatory properties. Their potent free radical-scavenging capacity significantly reduces oxidative stress, yielding multiple health benefits: enhancing immune defenses, slowing the progression of chronic diseases, promoting cardiovascular health, and demonstrating potential cancer-preventive effects [11, 36].

Purslane (*Portulaca oleracea* L.) is an annual succulent herb belonging to the Caryophyllales order and Portulacaceae family. It can survive in a variety of adverse environments, including drought, salinity, and poor soils, demonstrating remarkable adaptability, resulting in its widely distributed throughout the world. The plant’s leaves, stems, roots, flowers, and seeds display five characteristic colors—green, red, white, yellow, and black—which correspond to the Five Elements theory (Wu Xing) in traditional Chinese culture, earning it the poetic name “Five-Element Vegetable”. This unique chromatic diversity not only reflects the plant’s adaptive evolution to its environment, but also perfectly embodies traditional Chinese medicinal concepts of “five colors nourishing five organs” [13] and “medicinal food homology” [20]. In nature, purslane exhibits remarkable intraspecific diversity, with many cultivars showing significant variations in morphology and coloration. Beyond stem color variations, certain cultivars display red leaf phenotypes, and in some cases, even exhibit differential coloration between adaxial and abaxial leaf surfaces. However, the molecular mechanisms underlying the purple-red pigmentation of purslane stems and leaves remain incompletely understood. Notably, as a typical C4 plant, its pigment biosynthesis and regulatory networks may involve relatively complex mechanisms [45]. The prevailing hypothesis holds that in plants of the order Caryophyllales, anthocyanins and betalains are mutually exclusive—that is, plants that produce betalain pigments consistently lack anthocyanins, and vice versa [6, 41]. The reason is that, during the evolution of Caryophyllales, some key genes involved in anthocyanin biosynthesis—such as *Transparent Testa 19* (*TT19*)—have undergone repeated and complete loss in betalain-producing plants [30]. Although most other structural and regulatory genes related to flavonoid biosynthesis remain conserved in these lineages, the systematic absence of the *TT19* gene has resulted in the permanent loss of anthocyanin biosynthesis in betalain-producing lineages. It seems that the purple-red coloration observed in purslane is mainly due to the accumulation of betalains rather than anthocyanins. However, Sajiv et al. [33] proposed that anthocyanins are present in purslane, and our study further supports and confirms this view, demonstrating that purslane can not only synthesize anthocyanins but also accumulate them. Nevertheless, the specific biosynthetic pathway and regulatory mechanisms of anthocyanins in purslane remain to be elucidated.

To further explore the molecular mechanisms underlying the purple-red coloration of purslane stems and leaves, we selected three cultivars with different stem and leaf colors and conducted comprehensive non-targeted metabolomics and transcriptomics sequencing. Through integrated analyses, we characterized the key genes causing the color differences. Meanwhile, we systematically identified the glutathione S-transferase (GST) gene family and investigated its potential contribution to anthocyanin biosynthesis in purslane. These research findings enrich our understanding of pigment metabolism in Portulaca and provide a theoretical basis for the molecular improvement of color traits in C4 plants.

## Results

### Color variation and pigment accumulation among three cultivars of *P. oleracea*

**Fig. 1:**
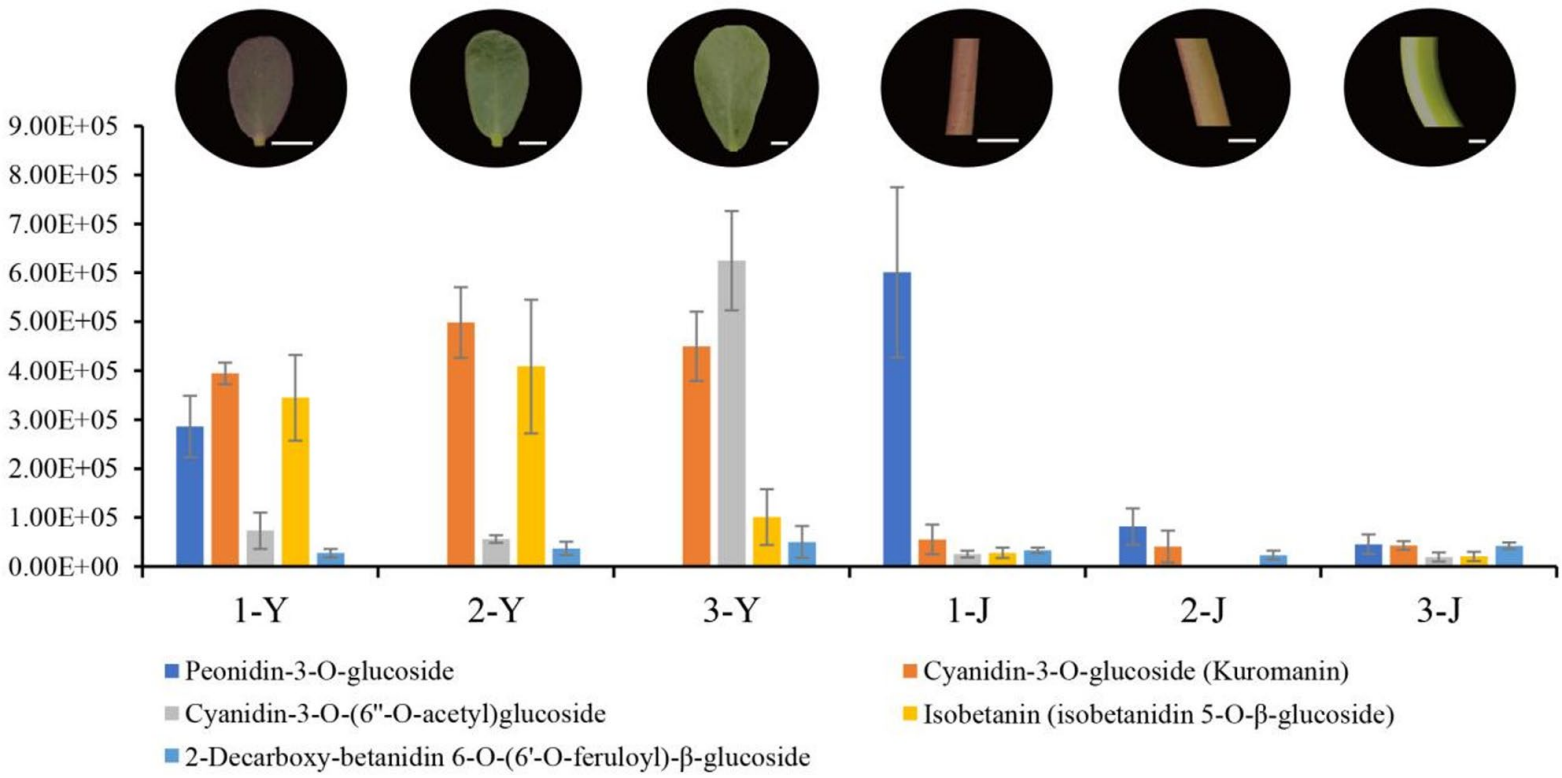
Differential accumulation of anthocyanins and betalains in leaves (Y) and stems (J) of *P. oleracea* with distinct color phenotypes. Bar chart shows the relative contents of five representative pigments, including anthocyanins (Peonidin-3-O-glucoside, Cyanidin-3-O-(6’’-O-acetyl)glucoside, Cyanidin-3-O-glucoside) and betalains (2-Decarboxy-betanidin 6-O-(6’-O-feruloyl)-β-glucoside, Isobetanin). “1”, “2”, and “3” refer to three different cultivars. Photographs above each column represent the corresponding leaf or stem color phenotype. Scale bar = 1 cm

The three cultivars of *P. oleracea* show distinct phenotypes: cultivar 1 has smaller leaves and thinner stems, both of which are red; cultivar 2 has medium-sized leaves and stems, with green leaves and light red stems; cultivar 3 has the largest and thickest leaves, the thickest stems, and both the leaves and stems are green. Metabolomic analysis revealed distinct patterns of anthocyanin and betalain accumulation among the three *P. oleracea* cultivars (Fig. 1; Table S1). A total of three anthocyanins—Peonidin-3-O-glucoside, Cyanidin-3-O-glucoside, and Cyanidin-3-O-(6’’-O-acetyl)glucoside—and two betacyanins—Isobetanin and 2-Decarboxy-betanidin 6-O-(6’-O-feruloyl)-β-glucoside—were identified. Marked differences in pigment composition and abundance were observed among the cultivars and between leaves and stems. In leaves, both anthocyanins and betalains accumulated at relatively high levels. The 1-Y samples exhibited the highest contents of Peonidin-3-O-glucoside, Cyanidin-3-O-glucoside, and Isobetanin, corresponding to their purplish-red leaf color phenotype. In 2-Y samples, Cyanidin-3-O-glucoside and Isobetanin were the predominant pigments, whereas in 3-Y samples, Cyanidin-3-O-glucoside and Cyanidin-3-O-(6’’-O-acetyl)glucoside were more abundant. In stems, the overall pigment levels were much lower than those in the leaves. The 1-J samples showed the highest concentration of Peonidin-3-O-glucoside, consistent with their purplish-red stem phenotype, while both anthocyanins and betalains were present in relatively small quantities in 2-J and 3-J samples. Overall, among all detected pigments, only the accumulation pattern of Peonidin-3-O-glucoside was highly consistent with the color variation of leaves and stems, suggesting that this compound may be the key pigment determining the coloration differences in *P. oleracea*.

### Metabolite analysis on three cultivars of *P. oleracea*

**Fig. 2:**
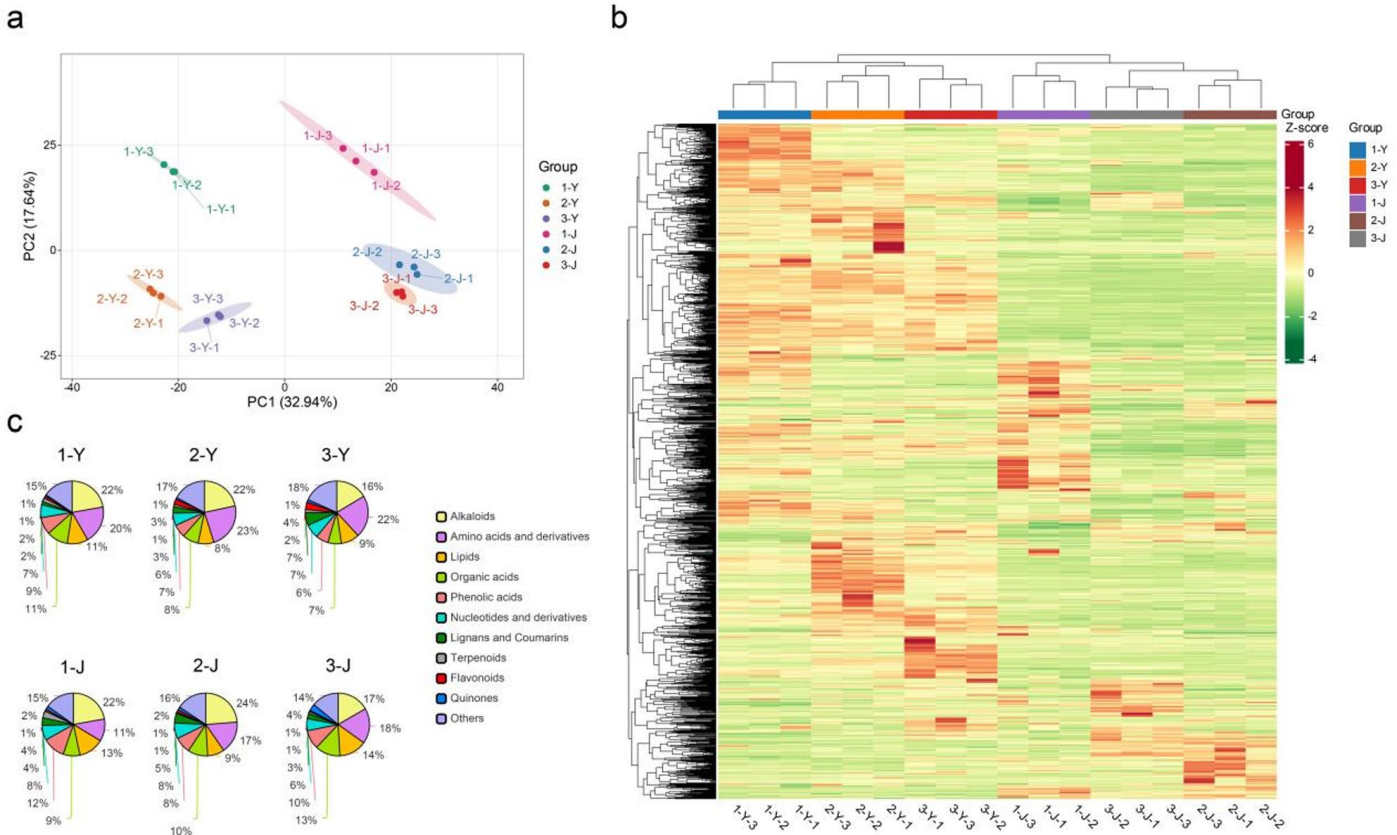
Overview of metabolomic profiles in leaves and stems of the three cultivars. Numbers 1, 2, and 3 represent three different cultivars of *P. oleracea*; Y indicates leaf samples and J indicates stem samples. **a** PCA of six groups based on metabolite profiles. **b** Heatmap and hierarchical clustering analysis of metabolite abundance, where each row represents a metabolite and each column represents a sample; colors indicate Z-score standardized abundance levels. **c** Relative abundance distribution of different metabolite categories in representative samples from each group

To compare the differences in color-related metabolites among three cultivars of *P. oleracea*, 18 samples’ metabolomics data from two tissues of three cultivars were obtained and analyzed. In this work, correlation analysis showed that the correlation between replicate samples was generally close to 1, indicating good consistency in the measurement results of leaf and stem traits within the same cultivar (Fig. S1). Principal component analysis (PCA) further confirmed that there was a clear separation between different groups and strong aggregation within the same group, demonstrating the high reliability of the obtained data (Fig. 2a). Hierarchical clustering analysis revealed distinct clustering patterns in the Z-score distributions of metabolites, demonstrating significant variations between different organs (leaves vs. stems) and among various cultivars (Fig. 2b). These findings clearly indicated that both genetic background (cultivar) and tissue type (organ) exert substantial influences on the global metabolite expression profiles.

Analysis of the overall metabolite composition indicated that amino acids and their derivatives, together with alkaloids, were the predominant compounds among all samples (Fig. 2c; Table S2). Visibly, metabolite distributions from the same tissue showed unique variations among cultivars. For example, in leaves, sample 1-Y possessed higher levels of phenolic acids, organic acids, demonstrating a moderate accumulation advantage; in stems, 1-J exhibited the highest enrichment of terpenoids among all samples, while 3-J stood out for its accumulation of quinones. Notably, the content of amino acids and their derivatives in all three leaf samples (1-Y, 2-Y, 3-Y) was higher than in their corresponding stem samples (1-J, 2-J, 3-J), indicating a clear advantage for leaves in accumulating these substances. Overall, distinct accumulation patterns were observed among metabolite categories across the three cultivars, with clear differences in relative abundance of major metabolites metabolites between leaves and stems.

**Fig. 3:**
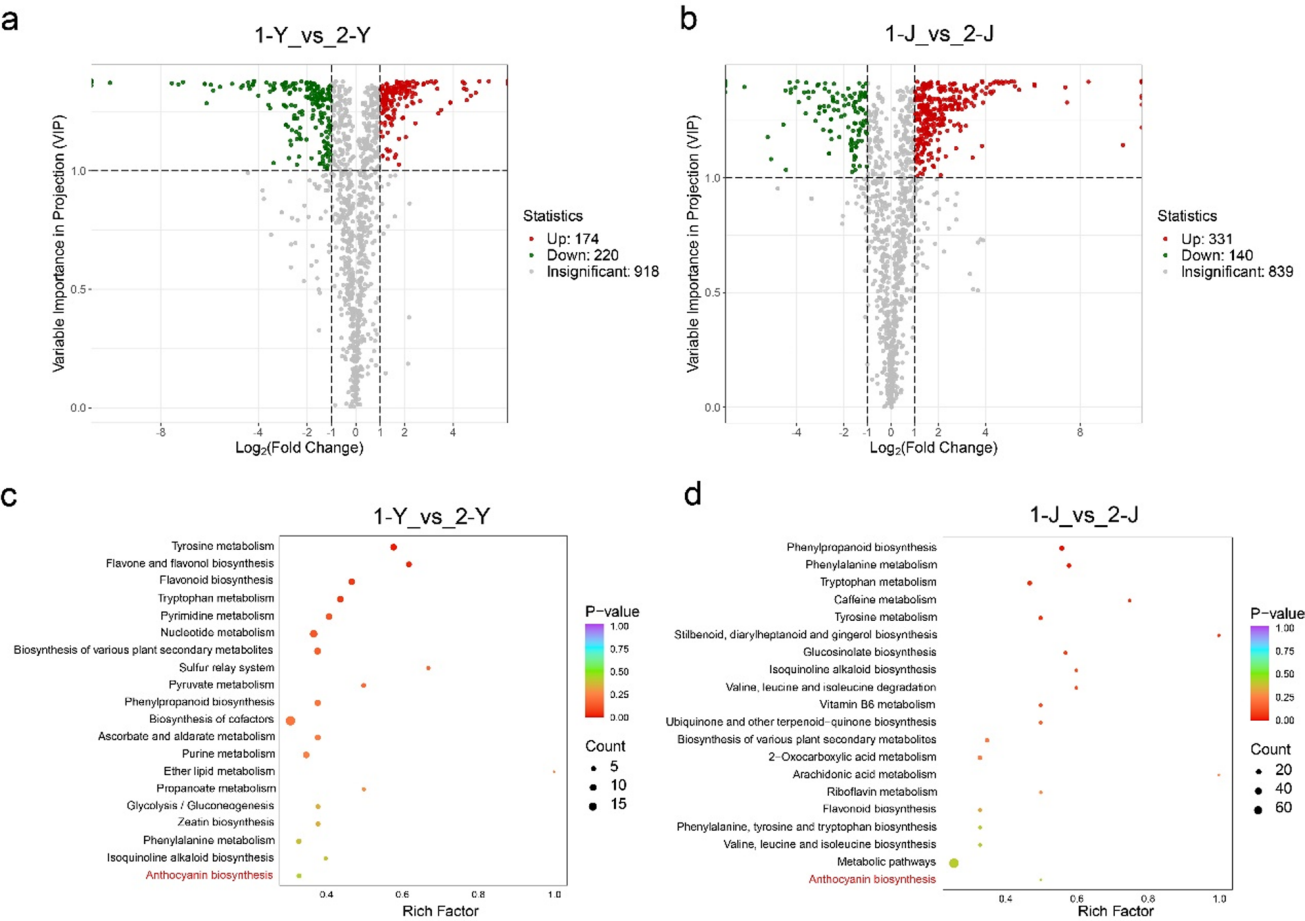
Differential metabolite and pathway enrichment analysis in leaves and stems between cultivar 1 and 2. Panels (**a**) and (**b**) show volcano plots of differential metabolites in leaves (1-Y vs. 2-Y) and stems (1-J vs. 2-J), respectively, where red and green dots indicate significantly up- and downregulated metabolites, and grey dots represent non-significant changes. **c** and (**d**) Top 20 KEGG enrichment bubble plots of differential metabolites in leaves and stems, where the y-axis indicates pathway names, the x-axis indicates the enrichment factor, bubble size represents the number of differential metabolites, and color indicates *p*-value significance

To comprehensively elucidate the metabolic differences among different cultivars, we performed multiple comparative analyses of metabolite profiles in leaves (Y) and stems (J). Volcano plot analysis showed that, in the comparison between 1-Y and 2-Y, 174 metabolites were upregulated and 220 were downregulated (Fig. 3a); whereas in the comparison between 1-J and 2-J, 331 metabolites were upregulated and 140 were downregulated (Fig. 3b). KEGG enrichment analysis showed that the differential metabolites in leaves were mainly enriched in pathways such as tyrosine metabolism, flavone and flavonol biosynthesis, and flavonoid biosynthesis, while those in stems were primarily associated with phenylpropanoid biosynthesis, phenylalanine metabolism, and tryptophan metabolism. Additionally, the anthocyanin biosynthesis pathway was significantly enriched in both leaves and stems, respectively, which might be a key factor influencing the coloration of different between cultivar 1 and 2 (Fig. 3c and d).

In the comparative analyses of other groups, including 1-Y vs. 3-Y, 1-J vs. 3-J, 2-Y vs. 3-Y, and 2-J vs. 3-J, each group exhibited varying numbers of upregulated and downregulated metabolites (Fig. S2 and 3). Further KEGG enrichment analysis indicated that the top 20 enriched pathways mainly included primary carbohydrate metabolism, amino acid metabolism, lipid metabolism, and the biosynthesis of some secondary metabolites, while no significant enrichment was observed in anthocyanin biosynthesis or related pathways. However, when the scope was expanded to the top 50 enriched pathways, the anthocyanin biosynthesis pathway began to appear in the 1-Y vs. 3-Y and 1-J vs. 3-J groups. This might be related to the formation of color differences between cultivar 1 and 3.

Although only Peonidin-3-O-glucoside was enriched in the anthocyanin biosynthesis pathway, considering that only 3 anthocyanin compounds were identified in this study (Fig. 1; Table S1), Peonidin-3-O-glucoside is likely the key metabolite influencing the phenotypic differences in *P. oleracea*. Further analysis revealed significant differences in peonidin-3-O-glucoside content among the three cultivars and between two tissues (Table S3). Cultivar 1 exhibited the highest peonidin-3-O-glucoside content in both leaves and stems, with especially pronounced accumulation in the stem tissue. Cultivar 2 had intermediate levels of peonidin-3-O-glucoside in stems, while cultivar 3 showed the lowest content. Overall, stems generally contained higher levels of peonidin-3-O-glucoside than leaves, and the accumulation in cultivar 1 was significantly higher than that in the other two cultivars (Fig. 1). Combined with phenotypic analysis, we found that the peonidin-3-O-glucoside levels in red tissues (1-Y, 1-J, 2-J) were significantly higher than those in green tissues (2-Y, 3-Y, 3-J), suggesting the accumulation of peonidin-3-O-glucoside is the main factor causing color differences among three cultivars of *P. oleracea*.

### Transcriptome profiling of three cultivars of *P. oleracea*

**Fig. 4:**
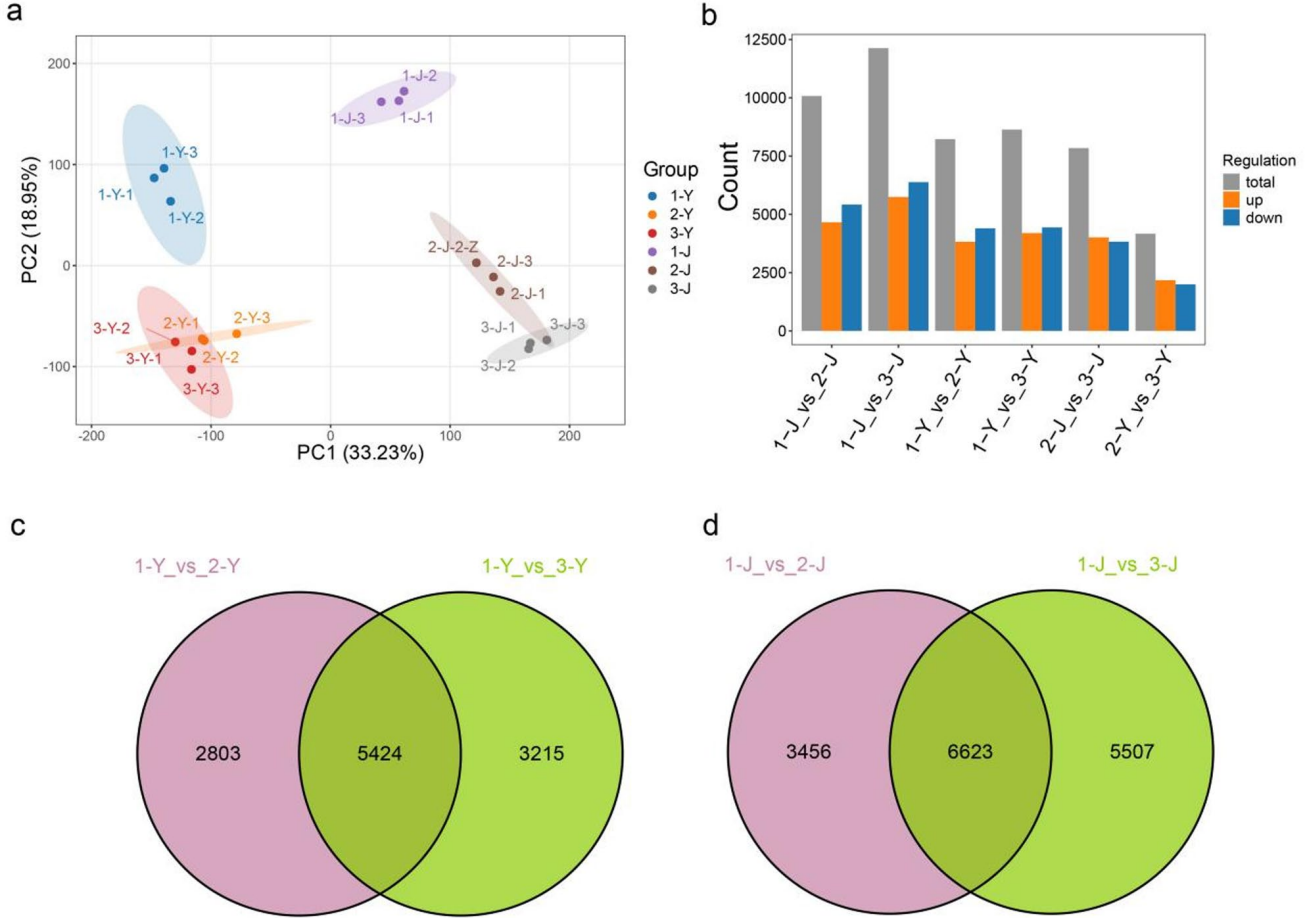
Transcriptome analysis results of different groups in leaves and stems among three cultivars. **a** PCA of the six groups based on transcriptome data; (**b**) Statistics of DEGs between groups; (**c**) and (**d**) Venn diagrams of DEGs between different leaf groups and stem groups, showing the overlap and unique DEG numbers

The quality control assessment of the transcriptome data demonstrated that, following filtration, approximately 47.82–71.15 million high-quality clean reads were obtained per sample, with GC contents ranging from 47.3% to 48.1% and Q30 values consistently exceeding 93.16% (Table S4). These results indicate that the sequencing data are of high quality and are well-suited for subsequent downstream analyses. Correlation analysis of transcriptome was consistent with those of the metabolomic correlation analysis, indicating good consistency between replicate samples (Fig. S4). The PCA results showed that samples from the six groups were clearly separated along the PC1 and PC2 components (Fig. 4a). Different cultivars and tissues formed different clusters in the principal component space. Samples within each group were tightly clustered, and there was clear separation between groups.

To clarify the molecular mechanism of anthocyanin synthesis in three different cultivars of *P. oleracea*, transcriptomic analysis was conducted to identify differentially expressed genes (DEGs) in six groups. The total number of DEGs was highest in the 1-J_vs_3-J comparison, followed by 1-J_vs_2-J, while 2-Y_vs_3-Y had the lowest number of differential genes. In each comparison, the numbers of upregulated and downregulated genes were approximately equal (Fig. 4b). In the leaf group, there were 5,424 DEGs shared between 1-Y_vs_2-Y and 1-Y_vs_3-Y, with 2,803 DEGs unique to 1-Y_vs_2-Y and 3,215 DEGs unique to 1-Y_vs_3-Y (Fig. 4c). Additionally, 1-Y_vs_2-Y and 2-Y_vs_3-Y shared 1,992 DEGs, with 6,235 and 2,179 unique DEGs, respectively; whereas 1-Y_vs_3-Y and 2-Y_vs_3-Y shared 2,252 DEGs, with 6,387 and 1,919 unique DEGs, respectively (Fig. S5). In the stem group, 1-J_vs_2-J and 1-J_vs_3-J shared 6,623 DEGs, with 3,456 and 5,507 unique DEGs, respectively (Fig. 4d). Meanwhile, 1-J_vs_2-J and 2-J_vs_3-J had 3,589 DEGs in common, with 6,490 and 4,254 unique DEGs, respectively; 1-J_vs_3-J and 2-J_vs_3-J shared 4,458 DEGs, with 7,672 and 3,385 unique DEGs, respectively (Fig. S5).

### Identification and validation of key DEGs related to anthocyanin metabolism

**Fig. 5:**
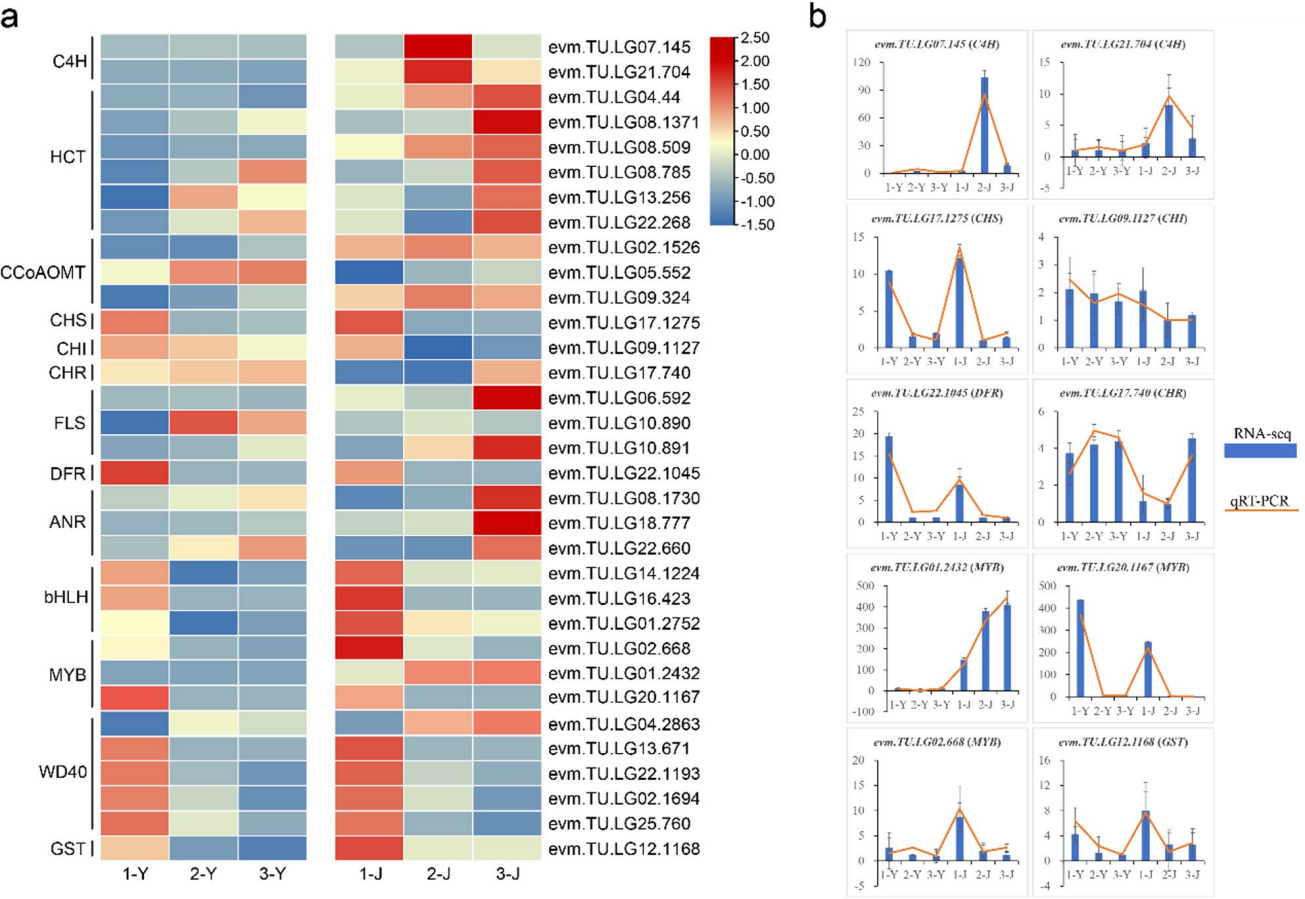
The expression levels of key genes involved in anthocyanin metabolism across two tissues of three cultivars. **a** Heatmap of the expression levels of key genes in different samples. The color gradient represents log_2_-transformed expression levels, with blue indicating low expression and red indicating high expression. **b** Validation of RNA-seq data using qRT-PCR for 10 DEGs. The y-axis denotes gene expression levels

To identify key regulatory genes involved in anthocyanin biosynthesis in *P. oleracea*, we grouped the samples based on their color characteristics: 1-Y, 1-J, and 2-J were classified as red, while 2-Y, 3-Y, and 3-J were classified as green. Genes that consistently showed differential expression trends in the comparisons of 1-Y vs. 2-Y, 1-Y vs. 3-Y, 1-J vs. 3-J, and 2-J vs. 3-J within the flavonoid and anthocyanin biosynthesis pathways were selected as candidate genes for further analysis. As a result, we identified 21 DEGs, among which five were upregulated and 16 were downregulated in red tissues (Fig. 5a; Table S5). These DEGs included two trans-cinnamate 4-monooxygenase (C4H) [EC:1.14.14.91], six shikimate O-hydroxycinnamoyltransferase (HCT) [EC:2.3.1.133], three caffeoyl-CoA O-methyltransferase (CCoAOMT) [EC:2.1.1.104], three flavonol synthase (FLS) [EC:1.14.20.6], three anthocyanidin reductase (ANR) [EC:1.3.1.77], as well as one each of chalcone synthase (CHS) [EC:2.3.1.74], chalcone isomerase (CHI) [EC:5.5.1.6], chalcone reductase (CHR), and bifunctional dihydroflavonol 4-reductase/flavanone 4-reductase (DFR) [EC:1.1.1.219/1.1.1.234]. In addition to these structural genes, we also identified 80 MYB, 117 bHLH, and 93 WD40 transcription factors (TFs) in genome-wide of *P. oleracea* (Table S6). By integrating transcriptomic and metabolomic data using nine-quadrant analysis, we ultimately identified 11 key TFs closely related to anthocyanin metabolism: 1 MYB associated with dihydroquercetin, 1 MYB and 1 WD40 associated with dihydrokaempferol, as well as 1 MYB, 3 bHLHs, and 4 WD40s related to peonidin. Among the TFs associated with peonidin, 2 bHLHs and 3 WD40s were also identified in the WGCNA results (Fig. 5a and S6; Tables S7 and 8).

Among the key genes identified, 6 HCT genes were highly expressed in 3-J, the CCoAOMT gene (*evm.TU.LG05.552*) was upregulated in 2-Y and 3-Y, and the CHR gene exhibited elevated expression in 2-Y, 3-Y, and 3-J. In addition, 3 FLS genes were highly expressed in 2-Y or 3-J, and 3 ANR genes were strongly expressed in 3-J. As all these genes encode negative regulators of anthocyanin accumulation, their high expression coincided with green coloration in the respective tissues. These findings suggest that these genes likely suppress anthocyanin biosynthesis in *P. oleracea* by diverting precursor flux away from anthocyanin formation and toward competing branches of the phenylpropanoid pathway, such as general flavonoid or lignin biosynthesis. In contrast, 2 C4H genes were highly expressed in 2-J, while CHS, CHI, DFR, and GST showed high expression in 1-Y and 1-J. These genes are recognized as positive regulators of anthocyanin accumulation, and the corresponding organs exhibited red pigmentation. This pattern indicates that these genes may promote anthocyanin accumulation in *P. oleracea* by upregulating key enzymes in the anthocyanin biosynthetic pathway, thereby enhancing pigment synthesis, stabilization, and intracellular transport, ultimately leading to visible coloration. Furthermore, 2 MYB, 3 bHLH, and 4 WD40 transcription factor genes were highly expressed in 1-Y or 1-J, suggesting that they may form the canonical MYB-bHLH-WD40 (MBW) transcriptional complex to positively regulate anthocyanin biosynthesis. In contrast, the MYB gene (*evm.TU.LG01.2432*) and the WD40 gene (*evm.TU.LG04.2863*) exhibited higher expression levels in 3-J, implying that they may act as negative regulators in specific tissues or developmental stages, potentially through a feedback mechanism that represses anthocyanin biosynthesis and leads to reduced pigment accumulation. To confirm the accuracy of the RNA-seq findings, 10 representative DEGs identified previously were subjected to quantitative real-time RT-PCR (qRT-PCR) validation. The qRT-PCR data revealed that the relative transcript abundance of these genes in the leaves and stems of the three *P. oleracea* cultivars showed a consistent trend with the RNA-seq results (Fig. 5b).

### Identification and phylogenetic analysis of the GST gene family

**Fig. 6:**
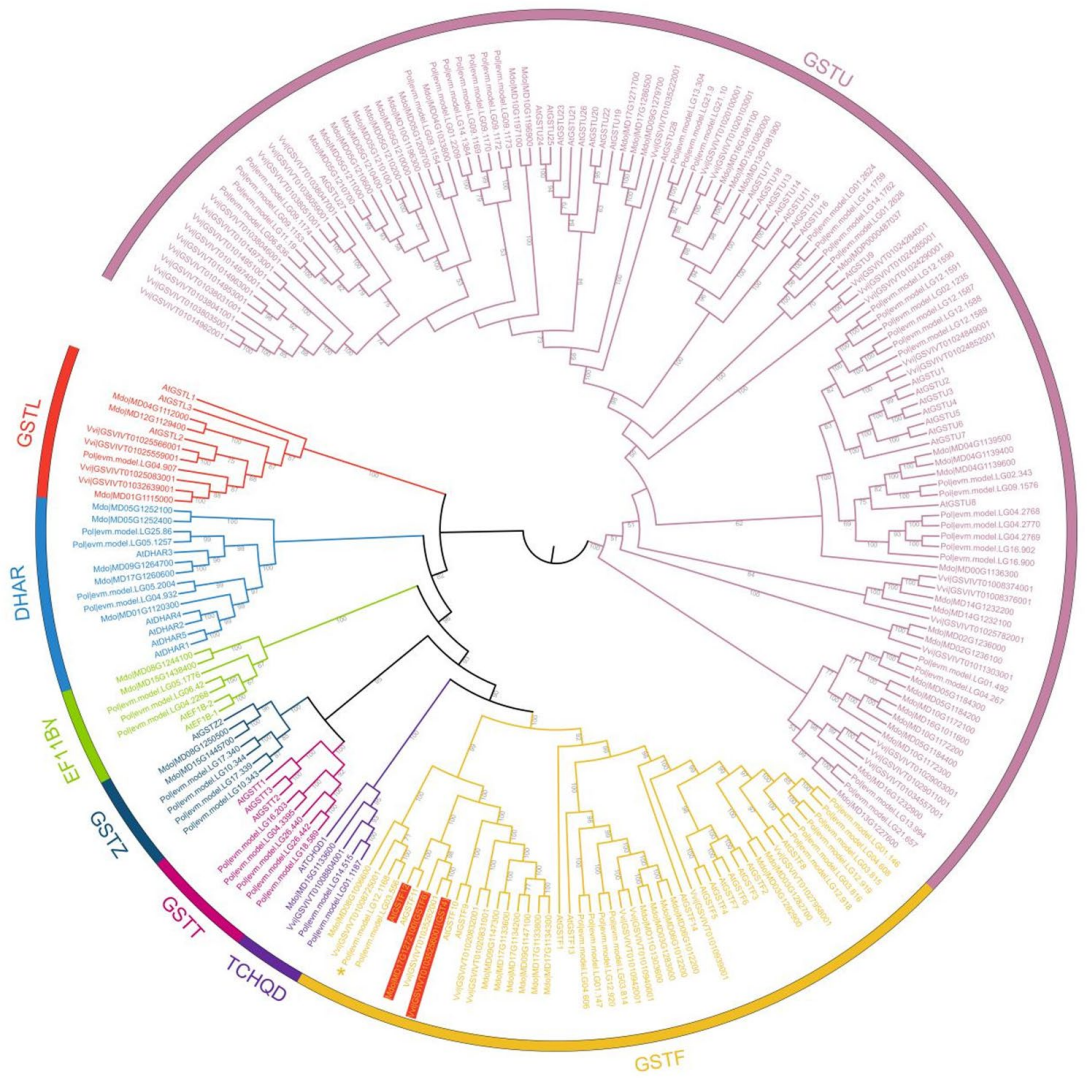
Phylogenetic tree constructed with GST protein sequences from *Arabidopsis thaliana* (55), apple (63), grape (42), and *P. oleracea* (61). Different colors represent different classes. Three functionally characterized *TT19* genes are highlighted with a red background. The potential functional gene in *P. oleracea* is marked with yellow star

*TT19* is a member of the Phi class (GSTF) within the plant *GST* gene family. It was first identified in *Arabidopsis* as *AtGSTF12* [17], where it plays a crucial role in the transport of anthocyanins and other flavonoids, thereby contributing significantly to pigment synthesis and accumulation. Orthologs of *TT19* in other species, such as *MdGSTF6* in apple [14] and *VvGST4* in grape [19], have also been shown to perform similar functions. Therefore, to determine whether a key *TT19* gene is present in *P. oleracea*, we performed a comprehensive identification and analysis of the *GST* gene family in this species. Phylogenetic analysis revealed that 61 *GST* genes can be classified into eight subclasses, consistent with previous studies [19] (Fig. 6; Table S9). Notably, in the GSTF class, three functionally characterized *TT19* genes—*AtGSTF12*, *MdGSTF6*, and *VvGSTF4*—clustered together, yet no *GST* genes from *P. oleracea* were found in this class. However, through the integration of expression profiling and nine-quadrant association analysis and WGCNA, we identified a *GST* gene (*evm.TU.LG12.1168*) that is positively correlated with Peonidin-3-O-glucoside biosynthesis (Fig. 5a; Table S10). Although *evm.TU.LG12.1168* does not cluster phylogenetically with the three functionally characterized *TT19* genes, it may perform a similar role in pigment synthesis and accumulation in *P. oleracea*.

### Integrated metabolomic and transcriptomic results to reveal anthocyanin metabolism in *P. oleracea*

**Fig. 7:**
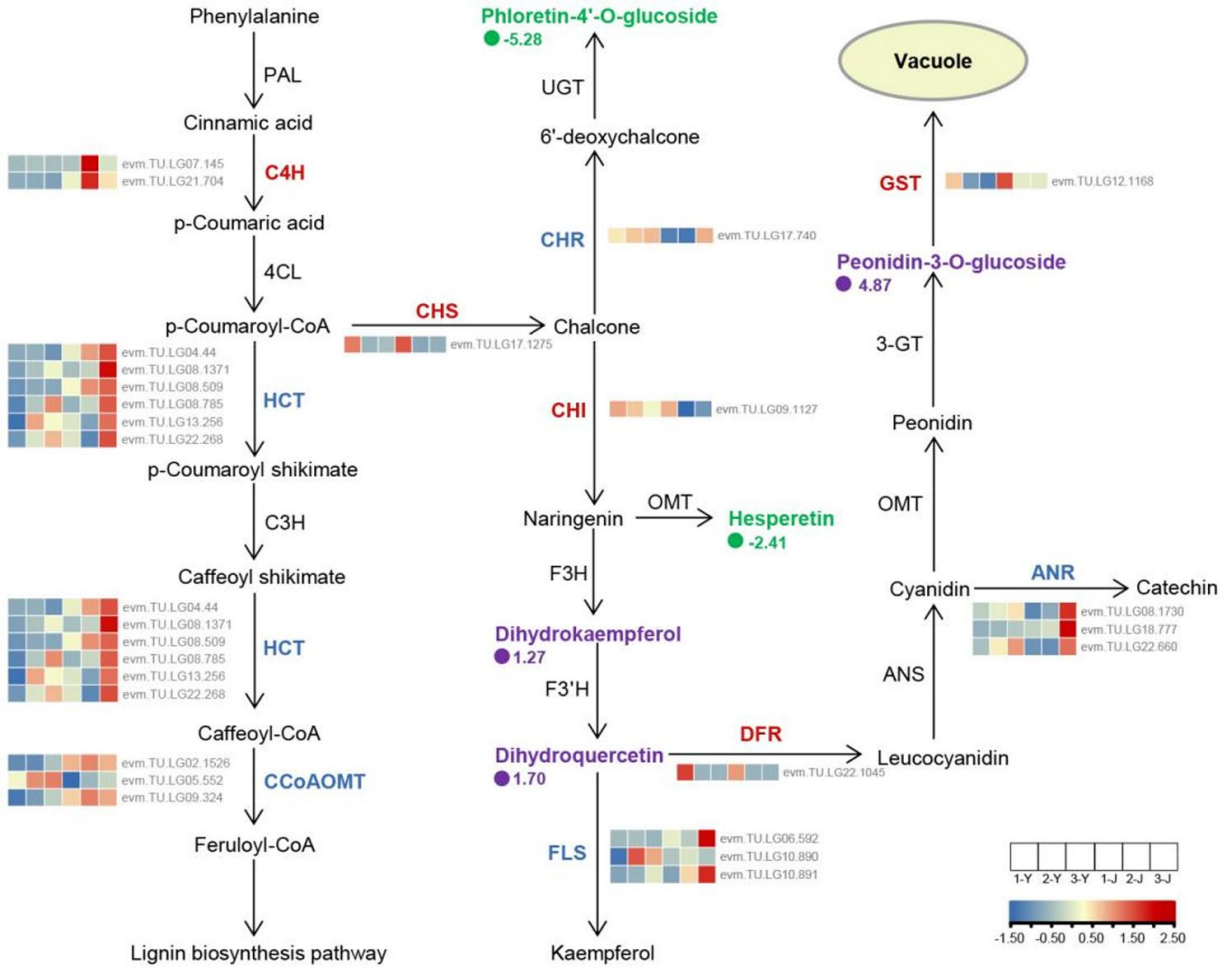
Metabolomics and transcriptomics analyses of paeoniflorin biosynthesis and related pathways in *P. oleracea*. Upregulated metabolites are marked in purple, downregulated metabolites are marked in green; upregulated enzymes are marked in red; downregulated enzymes are marked in blue. Colored squares represent gene expression levels, with expression data log_2_-transformed. The purple and green circles indicate the fold-change in metabolite levels, reflecting the relative change between the red tissues and green tissues of *P. oleracea*, with the data log_2_-transformed. ANS: Anthocyanidin synthesis; C3H: p-Coumaroyl-CoA 3-hydroxylase; F3H: Flavanone 3-hydroxylase; F3’H: flavanone 3’-hydroxylase; OMT: O-methyltransferase; PAL: phenylalanine ammonia-lyase; 3-GT: anthocyanidin 3-O-glucosyltransferase; 4CL: 4-coumarate-CoA ligase; UGT: UDP-Glucosyltransferase

Based on the analysis of key differential metabolites and DEGs, we constructed a model diagram of anthocyanin biosynthesis in *P. oleracea* (Fig. 7). The study indicates that the upregulation of *C4H*, *CHS*, and *CHI* promotes the accumulation of dihydrokaempferol and dihydroquercetin. Dihydroquercetin, catalyzed by the downstream upregulated enzyme *DFR*, further facilitates the increase of Peonidin-3-O-glucoside, a major component contributing to the redness of *P. oleracea*. Ultimately, Peonidin-3-O-glucoside is transported to the vacuole by *GST* (*evm.TU.LG12.1168*). Meanwhile, the downregulation of *CHR*, *FLS*, *ANR*, *HCT*, and *CCoAOMT*, along with the reduction of Phloretin-4’-O-glucoside and Hesperetin, further promotes the accumulation of anthocyanins. This is because the metabolic pathways involving these genes and metabolites all compete with the pathways leading to paeoniflorin synthesis.

## Discussion

*P. oleracea* is a widely distributed wild plant with strong environmental adaptability. Research shows that it is capable of maintaining robust growth under various adverse conditions such as salinity and drought, demonstrating remarkable stress resistance [22]. This adaptability largely stems from the efficient accumulation and dynamic regulation of metabolites related to osmotic adjustment and antioxidation [2]. Metabolomic analysis in this study shows that alkaloids, amino acids and derivatives, lipids, organic acids, and phenolic acids are the five metabolite categories with the highest relative abundance across all groups. It indicated that these core metabolites play vital roles in the primary physiological processes, including energy metabolism, structural construction, signal regulation, and stress defense. Although some fluctuations in proportion are observed among different groups, the stable enrichment of these main metabolite classes under various cultivars and tissues reflects the highly conserved and flexibly regulated nature of *P. oleracea*’s central metabolic network.

The unique presence of betalains in Caryophyllales plants has sparked intense scientific debate for over half a century regarding their evolutionary relationship with anthocyanins. Ehrendorfer [9] proposed that the ancestor of Caryophyllales originated in arid to semi-arid environments and primarily relied on wind pollination, which led to the loss of anthocyanin pigments. Later, as pollinators diversity increased and ecological environment changed, Caryophyllales transitioned to insect pollination and subsequently evolved betalains. In contrast, Clement and Mabry [6] postulated that the ancestors of Caryophyllales may have possessed both anthocyanins and betalains, but the extant lineages selectively lost one pigment or the other. Recent research has also provided support for these hypotheses [30]. However, an increasing number of studies have revealed that the presence of anthocyanins in some Caryophyllales plants. For example, Zhou et al. [48] compared red- and white-fleshed pitaya varieties and found that although betalains are the main pigments, several key genes in the anthocyanin biosynthetic pathway were significantly upregulated in red-fleshed samples, suggesting the pathway is not entirely lost. Furthermore, Dávila-Lara et al. [8] established that the red pigmentation in *Nepenthes* tissues originates from anthocyanins rather than betalains. As a member of Caryophyllales, *P. oleracea* has long been considered devoid of anthocyanins [25]. However, some researches confirmed that anthocyanins were present in the leaves, stems, and flowers of *P. oleracea* [7, 35]. In this study, a widely targeted metabolomics approach was used to systematically analyze the composition and content of anthocyanins in two tissues of three cultivars. The results showed that *P. oleracea* predominantly contains the peonidin-3-O-glucoside, and its distribution and content corresponded closely to color phenotypes. This indicates that the accumulation of peonidin-3-O-glucoside largely determines the color diversity of *P. oleracea*. It is worth noting that, except for the 2-J sample group, isobetanin was detected in all other sample groups based on the metabolomic data. This finding revises the previously held mutual exclusivity theory of betalains and anthocyanins in Caryophyllales plants. It should be noted that this study included only three cultivars, and the limited sample size may affect the broader applicability of the conclusions. In addition, we acknowledge that the quantitative accuracy of the widely targeted metabolomics method is limited. Consequently, our findings primarily serve to characterize the major pigment categories on a qualitative basis. In follow-up work, we will further expand the sample size to include more *P. oleracea* cultivars and combine targeted quantitative analysis techniques to accurately determine the accumulation levels of anthocyanins and betalains, thereby systematically elucidating the metabolic relationship and interaction mechanisms between them.

*TT19*, a member of *GST* gene family, play a key role in the transport and accumulation of anthocyanins in plants. In *Arabidopsis*, mutants of this gene exhibit a transparent seed coat and absence of anthocyanin, highlighting the crucial role in pigment accumulation [21, 38]. The importance of *TT19* for anthocyanin accumulation has also been confirmed in several crop species, including cotton [5], *Medicago truncatula* [44], poinsettia [42], and bayberry [46]. In some plants, such as tea (*Camellia sinensis*), members of the GSTU subclass (e.g., *CsGSTU18*) have been found to perform functions similar to *TT19* in anthocyanin accumulation and stem purpling [47]. In *P. oleracea*, we identified a *GST* gene closely related to anthocyanin synthesis, which we speculate may play a compensatory or supplementary role in the absence of *TT19*. The specific mechanism of its function requires further investigation. This is of great significance for elucidating the evolutionary and functional diversification of the plant pigment metabolic network and for understanding the adaptive evolution of higher plants.

In summary, this study is the first to systematically reveal the compositional characteristics of metabolites in purslane through integrated multi-omics analysis, and to elucidate the molecular mechanisms underlying its anthocyanin biosynthesis. These findings not only correct the traditional belief that betalain-producing plant groups are incapable of synthesizing anthocyanins, but also provide new evidence for understanding the complexity of pigment metabolic networks. The results provide a theoretical foundation and genetic resources for the development of functional foods using purslane and for in-depth studies of related molecular mechanisms, and also open up new research avenues in pigment evolution and molecular breeding within the Caryophyllales.

## Conclusions

This study identified alkaloids, amino acids and derivatives, lipids, organic acids, and phenolic acids as the five most abundant classes of metabolites in *P. oleracea*. The results showed that peonidin-3-O-glucoside is the main anthocyanin, and its accumulation in different organs is closely related to color variation. Meanwhile, 21 key structural genes and 11 related TFs involved in anthocyanin biosynthesis were identified. Further expression pattern analysis systematically revealed the molecular regulatory mechanisms underlying anthocyanin biosynthesis in *P. oleracea*. In addition, the results indicated that one member of *GST* gene family, *evm.TU.LG12.1168*, may compensate for the absence of the *TT19* gene and play an important role in anthocyanin transport in *P. oleracea*. These findings not only deepen our understanding of the biosynthesis and regulatory mechanisms of pigments in *P. oleracea*, but also provide an important theoretical foundation and new research directions for studies on pigment metabolism and molecular breeding in Caryophyllales plants.

## Methods

### Cultivation and collection of plant materials

Three cultivars of purslane were cultivated and collected at the Yanqing experimental base of the Vegetable Research Center, Beijing Academy of Agricultural and Forestry Sciences. In 2024, mature leaf and stem tissues from these three purslane cultivars were sampled. Stem tissues were collected from the upper-middle section of the stems, with each segment approximately 2 cm in length. The experiment employed three independent biological replicates per sample, with each replicate consisting of three individual plants. From each plant, one mature leaf and one stem segment were collected.

### Metabolite analysis via UPLC-MS/MS

Identification and analysis of purslane metabolites were performed using UPLC-MS/MS [43]. The raw mass spectrometry data obtained were subsequently used for qualitative and quantitative analysis of metabolites, followed by statistical analysis as described below: Principal component analysis (PCA) was performed using the prcomp function in R, with data standardized by unit variance scaling prior to analysis. Hierarchical cluster analysis (HCA) and calculation of Pearson correlation coefficients (PCC) were both conducted using the R package ComplexHeatmap. HCA results were displayed as heatmaps with dendrograms to illustrate sample and metabolite clustering, while PCC results were presented as heatmaps showing correlations between samples. For HCA, normalized metabolite signal intensities (unit variance scaling) were visualized using a color spectrum. For two-group comparisons, differential metabolites were identified based on a variable importance in projection (VIP) score greater than 1 (VIP > 1) and an absolute log_2_ fold change (|Log₂FC| ≥ 1). VIP values were obtained from orthogonal partial least squares discriminant analysis (OPLS-DA), which also generated score plots and permutation tests, all implemented using the R package MetaboAnalystR. Prior to OPLS-DA, data were log_2_ transformed and mean-centered, and 200 permutations were conducted to prevent overfitting. Metabolites were annotated using the KEGG Compound database, and the annotated metabolites were mapped to the KEGG Pathway database. Pathways containing significantly regulated metabolites were further analyzed with metabolite set enrichment analysis (MSEA), and pathway significance was determined using *p*-values from the hypergeometric test.

### RNA extraction and sequencing and data analysis

Total RNA was extracted using a combination of ethanol precipitation and the CTAB-PBIOZOL method. The CTAB extraction buffer contained cetyltrimethylammonium bromide (CTAB; Sangon Biotech, Shanghai, China), polyvinylpyrrolidone (PVP-40; Amresco, USA), sodium chloride (Sinopharm, China), Tris-HCl (pH 8.0; Thermo Fisher Scientific, USA), and EDTA (pH 8.0; Thermo Fisher Scientific, USA). During extraction and purification, β-mercaptoethanol (Fuchen, Tianjin, China), phenol (Dingguo, Beijing, China), chloroform (Sinopharm, China), and lithium chloride (Sangon Biotech, Shanghai, China) were added to facilitate RNA isolation. DNA contamination was removed using DNase I and its reaction buffer (New England Biolabs, USA). The purified RNA was precipitated with absolute ethanol, dissolved in DEPC-treated water (Thermo Fisher Scientific, USA), and assessed for concentration and integrity using a Qubit fluorometer and a Qsep400 high-throughput fragment analyzer. Poly(A)-tailed mRNA was enriched using the Hieff NGS^®^ mRNA Isolation Master Kit V2 (Yeasen Biotechnology, Shanghai, China). Library construction was performed with the Hieff NGS^®^ Ultima Dual-mode mRNA Library Prep Kit for Illumina^®^ (Yeasen Biotechnology) according to the manufacturer’s instructions. First-strand cDNA was synthesized using random hexamer primers, and second-strand cDNA synthesis was conducted by substituting dUTP for dTTP to ensure strand specificity, followed by end repair and dA-tailing. Sequencing adapters were then ligated, and 250–350 bp fragments were selected and purified using magnetic beads. The resulting libraries were amplified by PCR, further purified, and subjected to quality assessment. Libraries that passed quality control were pooled according to effective concentrations and sequenced on an Illumina high-throughput platform with 150 bp paired-end reads. Fluorescence signals were captured and processed computationally to obtain sequencing data.

FastQC (http://www.bioinformatics.babraham.ac.uk/projects/fastqc/) was used to filter out adapters and low-quality sequences from the raw data. Then, the clean reads were mapped to the reference genome using HISAT v.2.2.1 [16] with default parameters. Novel transcript prediction and assembly were conducted using StringTie v.2.0 [28], whose network flow algorithm and optional de novo assembly enable the reconstruction of more complete and accurate transcripts. Gene expression levels were quantified using featureCounts, and FPKM values (fragments per kilobase of transcript per million mapped reads) were calculated based on gene length. Differential expression analysis between groups was performed with DESeq2 v.1.26.0 [23], with *P*-values adjusted using the Benjamini & Hochberg method; adjusted *P*-values and log_2_ fold-change were used as thresholds for defining significantly differentially expressed genes. The weighted gene co-expression network analysis (WGCNA) for transcriptomic data was performed using the WGCNA R package (Pei, Chen, & Zhang [27]),. Modules were detected using default settings with a mergeCutHeight of 0.25 and a minModuleSize of 50. One representative hub gene was selected from each module.

### qRT-PCR validation

To verify the reliability of the transcriptomic results, ten representative DEGs were selected for validation by quantitative real-time qRT-PCR. Primer pairs specific to each gene were designed using Premier 5 software. Each qRT-PCR reaction had a total volume of 20 µl, comprising 1 µl of cDNA template, 1 µl each of the forward and reverse primers, 10 µl of 2 × Taq Pro Universal SYBR qPCR Master Mix (Vazyme Biotech Co., Ltd.), and 7 µl of nuclease-free water. The cycling protocol consisted of an initial denaturation step at 95 °C for 30 s, followed by 40 cycles of 95 °C for 5 s, 60 °C for 15 s, and 72 °C for 20 s, using a Bio-Rad CFX96 Real-Time PCR Detection System (Bio-Rad, USA). Each gene was analyzed in triplicate to ensure technical reproducibility. The relative expression levels were determined following the 2⁻^ΔΔCT^ method [31], with β-tubulin serving as the internal reference gene.

### Identification and phylogenetic analysis of transcription factor gene family

To identify transcription factor gene family members of purslane, we performed BLASTP analysis using well-annotated genes from the previous studies as queries and conducted profile hidden Markov model (HMM) searches using domains (MYB, PF00249; bHLH, PF00010; WD40, PF00400; GST, PF00043 and PF02798) from the Pfam database [10] as seeds. These analyses were carried out against the genome-wide amino acid sequences in purslane [45], utilizing BLASTP (Camacho et al. 2009) (E-value < 10 − 5) and hmmsearch in HMMER v3.3 [29] (--domE 0.001) respectively. The final set of homologous genes was derived from the intersection of the two methods’ results. To construct the phylogeny of *GST* gene family, amino acid sequences were initially aligned using MAFFT v7.312 [15]. The corresponding nucleotide sequences were then aligned to fit the amino acid alignment using PAL2NAL v14 [39]. Poorly aligned regions were removed with trimAL v3 [4] using the parameter “-gt 0.3”. The phylogenetic tree was constructed using maximum likelihood in RAxML v8.2.12 [37] under the “GTRGAMMA” model with 1000 bootstrap replicates, and the algal sequence was used as the outgroup. The topology of the consensus trees was visualized using iTOL [18].

## Supplementary Information

Supplementary Material 1.

Supplementary Material 2.

## Data Availability

The raw transcriptome sequencing data have been deposited in the NCBI Sequence Read Archive (SRA) under BioProject PRJNA1299154 (https://www.ncbi.nlm.nih.gov/sra/PRJNA1299154). Other datasets supporting this study will be made available on request.

## Abbreviations

qRT-PCR: Quantitative real-time RT-PCR
TT19: Transparent Testa 19
DEGs: Differentially expressed genes
KEGG: Kyoto Encyclopedia of Genes and Genomes
PCA: Principal component analysis
PCC: Pearson correlation coefficients
C4H: Trans-cinnamate 4-monooxygenase
HCT: Shikimate O-hydroxycinnamoyltransferase
CCoAOMT: Caffeoyl-CoA O-methyltransferase
FLS: Flavonol synthase
ANR: Anthocyanidin reductase
CHS: chalcone synthase
CHI: Chalcone isomerase
CHR: Chalcone reductase
DFR: Dihydroflavonol 4-reductase
ANS: Anthocyanidin synthesis
C3H: p-Coumaroyl-CoA 3-hydroxylase
F3H: Flavanone 3-hydroxylase
F3’H: Flavanone 3’-hydroxylase
OMT: O-methyltransferase
PAL: pPhenylalanine ammonia-lyase
3-GT: Anthocyanidin 3-O-glucosyltransferase
4CL: 4-coumarate-CoA ligase
UGT: UDP-Glucosyltransferase
GST: Glutathione S-transferase
