# Supplementary material for: Integrated metabolome and transcriptome analysis provides insights into anthocyanin biosynthesis in Portulaca oleracea
Source: BMC Plant Biol. 2025 Nov 27;25:1774. doi: 10.1186/s12870-025-07790-2 (PMC12752247; doi:10.1186/s12870-025-07790-2)
Supplement: Supplementary file 2 — Supplementary Material 2. [file 12870_2025_7790_MOESM2_ESM.docx]

**Supplementary Information**

**Supplementary Material 1:** Fig. S1. Correlation analysis of metabolite abundances in stems and leaves of three cultivars of *P. oleracea*. Each cell in the matrix shows the Pearson correlation coefficient between two samples, with the color intensity reflecting the strength of correlation. Numbers 1, 2, and 3 represent different cultivars (cultivar 1, 2, and 3); ‘Y’ denotes leaf samples, and ‘J’ denotes stem samples.

**Supplementary Material 2:** Fig. S2. Differential metabolite and pathway enrichment analysis between cultivar 1 and cultivar 3. a: Volcano plot of metabolite differences in leaves between cultivar 1 and cultivar 3 (1-Y vs 3-Y). b: Volcano plot of metabolite differences in stems between cultivar 1 and cultivar 3 (1-J vs 3-J). Red dots indicate significantly upregulated metabolites; green, significantly downregulated; gray, no significant change. c: KEGG pathway enrichment bubble plot of differential metabolites in leaves (1-Y vs 3-Y). d: KEGG pathway enrichment bubble plot of differential metabolites in stems (1-J vs 3-J). Bubble size reflects the number of differential metabolites, and redder color indicates higher statistical significance (lower p-value).

**Supplementary Material 3:** Fig. S3. Differential metabolite and pathway enrichment analysis between cultivar 2 and cultivar 3. a: Volcano plot of metabolite differences in leaves between cultivar 2 and cultivar 3 (2-Y vs 3-Y). b: Volcano plot of metabolite differences in stems between cultivar 2 and cultivar 3 (2-J vs 3-J). Red dots indicate significantly upregulated metabolites; green, significantly downregulated; gray, no significant change. c: KEGG pathway enrichment bubble plot of differential metabolites in leaves (2-Y vs 3-Y). d: KEGG pathway enrichment bubble plot of differential metabolites in stems (2-J vs 3-J). Bubble size reflects the number of differential metabolites, and redder color indicates higher statistical significance (lower p-value).

**Supplementary Material 4:** Fig. S4. Correlation analysis of transcriptome expression profiles in stems and leaves of three cultivars of *P. oleracea*. Each cell in the matrix shows the Pearson correlation coefficient between two samples, with color intensity indicating the strength of the correlation. Numbers 1, 2, and 3 represent different cultivars (cultivar 1, 2, and 3); ‘Y’ denotes leaf samples, and ‘J’ denotes stem samples.

**Supplementary Material 5:** Fig. S5. Venn diagram comparison of DEGs in stems (J) and leaves (Y) among different cultivar groups. Each subplot represents the DEGs between two different comparison groups, with the overlapping areas indicating the shared DEGs.

**Supplementary Material 6:** Figure. S6. Module-trait relationships in *Portulaca oleracea* based on WGCNA using Peonidin-3-O-glucoside content as the phenotypic trait. Each row corresponds to a gene co-expression module identified by WGCNA, labeled by a unique color. The number in parentheses indicates the number of genes in each module. The color scale represents the correlation coefficient between the module eigengene and Peonidin-3-O-glucoside content, with red indicating positive correlation and blue indicating negative correlation. The numbers in each cell indicate the correlation coefficient and the corresponding p-value (in parentheses).

**Supplementary Material 7:** Table S1. Relative abundance of anthocyanins and betacyanins in leaf (Y) and stem (J) tissues of three cultivars of *P. oleracea*.

**Supplementary Material 8:** Table S2. Relative abundance of major metabolite categories in the leaves (Y) and stems (J) of three *P. oleracea* cultivars.

**Supplementary Material 9:** Table S3. Flavonoid components related to anthocyanin biosynthesis detected in the stems and leaves of three cultivars.

**Supplementary Material 10:** Table S4. Information on the RNA-seq data, which was obtained by calculating the average of three replicates.

**Supplementary Material 11:** Table S5. Information on structure genes related to anthocyanin metabolism.

**Supplementary Material 12:** Table S6. List of MYB/bHLH/WD40 gene famliy members in *P. oleracea*. Red font in the text indicates genes identified by WGCNA as being positively correlated with Peonidin-3-O-glucoside.

**Supplementary Material 13:** Table S7. Information on trancription factor related to anthocyanin metabolism. Red font in the text indicates genes identified by WGCNA as being positively correlated with Peonidin-3-O-glucoside.

**Supplementary Material 14:** Table S8. Connectivity statistics of genes in the brown module identified by WGCNA.

**Supplementary Material 15:** Table S9. List of the GST gene famliy members in *P. oleracea*. Red font in the text indicates genes identified by WGCNA as being positively correlated with Peonidin-3-O-glucoside.

**Supplementary Material 16:** Table S10. Information on GST related to anthocyanin metabolism.
